# Associations between ambient air temperature, low birth weight and small for gestational age in term neonates in southern Israel

**DOI:** 10.1186/s12940-018-0420-z

**Published:** 2018-11-09

**Authors:** Itai Kloog, Lena Novack, Offer Erez, Allan C. Just, Raanan Raz

**Affiliations:** 1 Department of Geography and Environmental Development, Ben-Gurion University of the Negev, P.O.B, 653 Beer Sheva, Israel; 2 Department of Public Health, Ben-Gurion University of the Negev, P.O.B, 653 Beer Sheva, Israel; 3 Department of Gynecology and Obstetrics, Soroka medical center, Beer Sheva, Israel; 4 Department of Preventive Medicine, Icahn School of Medicine at Mt Sinai, New York, NY USA; 5 Braun School of Public Health and Community Medicine, Faculty of medicine, the Hebrew University of Jerusalem, Jerusalem, Israel

**Keywords:** Temperature, Climate change, Birth outcomes, Low birth weight, Epidemiology, Environmental exposure

## Abstract

**Background:**

The increase in ambient temperatures (Ta) and emissions of greenhouse gases over the last century has focused attention on the effects of ambient temperatures on health outcomes. We aimed to investigate the association between Ta and the clinical measures of term low birth weight (tLBW) and small for gestational age (SGA) in singleton term infants using a decade of regional hospital data in southern Israel.

**Methods:**

We linked all births in Soroka University Medical Center in the southern district of Israel insured by Clalit Health Services with pregnancy Ta estimated by our novel hybrid spatio-temporally resolved prediction model. Logistic regression generalized additive models and general linear models were used, with either tLBW or SGA as the dependent variable, modeling entire pregnancy and trimester-specific Ta adjusting for seasonality, time trend, particulate matter, maternal age, gravidity, parity, ethnicity, sex, poverty index and population density.

**Results:**

The study population included 56,141 singleton term newborns, with 1716 (3.1%) cases of tLBW and 8634 (15.4%) cases of SGA**.** The average and the median Ta across the entire pregnancy were 19.9 (SD: 1.77, range: 14.6–24.9) degrees centigrade. The lowest Ta quartile (Ta = < 18.5) was associated with higher risk of tLBW (odds ratio = 1.33, 95%CI 1.11–1.58) while the highest Ta quartile (Ta > =21.3) was not significantly associated with tLBW (odds ratio = 1.17, 95%CI 0.99–1.38), in comparison to the two intermediate quartiles. When analyzing SGA as the dependent variable, the lowest Ta quartile was associated with significantly higher risk of SGA (odds ratio = 1.18, 95%CI 1.09–1.29) while the highest quartile was associated with significantly lower risk of SGA (odds ratio = 0.91, 95%CI 0.84–0.99) in comparison to the two intermediate quartiles.

**Conclusions:**

Our findings suggest that lower pregnancy Ta may increase the risk of tLBW and SGA, and higher pregnancy Ta may decrease the risk of SGA in singleton term infants in southern Israel.

## Introduction

The rise in temperatures over the last century and emissions of greenhouse gases has focused attention on the effects of increasing heat [1]. The effects of temperatures and extreme events on health outcomes have been extensively studied [2–6].

Many studies have shown that heat is related to various health outcomes including an increase in total and cause specific mortality [4, 7–12] as well hospital admissions, emergency room visits and ambulance calls [13, 14]. The vulnerability of human populations to extreme temperatures is a function of their sensitivity to the exposure, of the character, magnitude and rate of the climate extreme, and of the adaptation measures and actions in place. The association between temperature and mortality has been identified and described as a non-linear U-, J- or V-shaped function, with the lowest mortality rates recorded at moderate temperatures, rising progressively as temperatures increase or decrease [7, 10, 13]. Finally, these studies have shown that the effects of temperature vary geographically [15, 16] and depend on local climatic conditions and population characteristics such as demography, socioeconomic conditions and health status [3].

Among many susceptible groups, pregnant women, fetuses and children are particularly vulnerable to the physiologic stress of extreme temperatures and sudden temperature variation [17, 18]. Yet only in recent years, studies have started to examine the association between maternal exposure to extreme ambient air temperatures (Ta) and adverse pregnancy outcomes. Basu and colleagues [19] examined apparent temperature (a combination of temperature and relative humidity) with full-term low birth weight (tLBW) comparing 43,629 tLBW infants and 2,032,601 normal weight infants in California from 1999 to 2013. They presented 13% (95% confidence interval (CI): 4.1–22.7%) increased risk of tLBW per 10 °F with entire pregnancy mean apparent temperature above 55 °F (12.8 C).

Li and colleagues [20], examined the effects of ambient temperature at three trimesters of pregnancy on preterm birth and stillbirth, and evaluate the effect changes during 1994–2013. Their study presented results showing that both low and high temperatures at the 3rd trimester of pregnancy significantly increased the risk of preterm birth, with similar hazard ratios (HR) for low (HR = 1.21,95% CI: 1.16–1.27) and high (HR = 1.2, 95% CI: 1.16–1.26) temperatures in comparison with minimum-prevalence temperatures for each trimester. A number of studies have looked at seasonal patterns in pregnancy outcomes, and recently some studies have focused on the relationship between ambient temperature exposure at different time-windows during pregnancy and outcomes including preterm birth and birth weight [18, 21–23].

Extremes of both high [24–29] and low [30] temperatures, mainly estimated during the last month or days of pregnancy, have been associated with an increased risk of preterm birth. Similarly, lower birth weight has been associated with both higher [31–34] and lower [35] ambient temperature during specific trimesters of pregnancy. In contrast, a large study of more than 480,000 births found no association between preterm birth and a variety of factors including temperature, humidity and barometric pressure [36].

These conflicting results published to date on the relationship of Ta with birth outcomes may be due to several reasons. There could be differences between local climatic and geographical conditions of the study areas as well as different levels of acclimatization among the pregnant women across the populations [2]. One established reason could be attributed to differences in temperature exposure assessment [11, 32]. In addition, there is limited evidence for populations living in suburban and rural areas due to the limited temperature data available. Most of these studies have been carried out in urban areas primarily due to data availability in terms of exposure and the number of outcome counts, large enough to ensure statistical power. This may result in lower variability of the exposure and possibly bias in the estimates related to the exposure considered due to uncertainty in assessment of individual exposure levels [37].

While preterm birth and multiple births pregnancy are the major reasons for low birth weight (LBW), approximately 90% of newborns are singleton term infants. tLBW and small for gestational age (SGA) are two main clinical measure that define fetal growth restriction in term infants, a phenomenon that predicts various complications in early childhood and beyond, including perinatal mortality, impaired immune system, respiratory morbidity and neurobehavioral disorders [38, 39]. In this study we make use of novel Ta estimation from our spatio-temporally resolved satellite-based hybrid models in Israel [40] to study the association between Ta and clinical measures of birth weight in term infants - tLBW and SGA, using regional hospital data from 2004 to 2013. Using these models, we can overcome the common limitation of insufficiently detailed climate data – in space and time – for estimating robust exposure to temperature and examine its associations with tLBW and SGA. In addition, we study this question in unique geo-climatic conditions that vary widely across southern of Israel, including 4 known climate zones (Mediterranean climate, semi-arid climate, arid climate and extreme arid climate) with maximum temperature which can exceed 45 °C in extreme days. These climate conditions and high Ta are important for insights into the effects of temperature on birth outcomes in a changing climate with extreme weather events.

## Methods

### Study domain and population

**Fig. 1:**
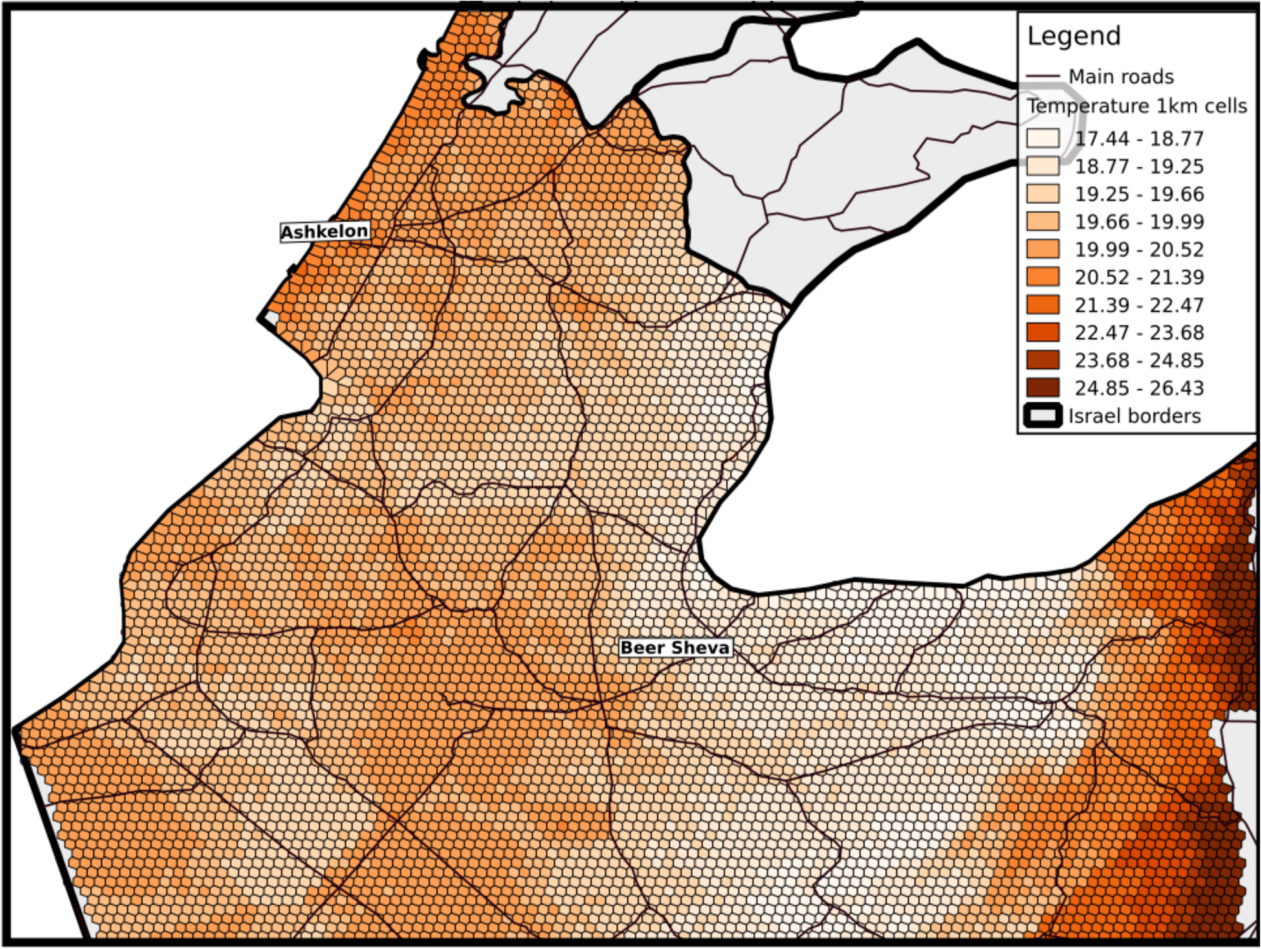
Mean temperature 1 km grids across the study area aggregated for the years 2004–2013

The research area is the Southern district of Israel (see Fig. 1). The research area has unique geo-climatic conditions that vary widely across Southern of Israel, including 4 known climate zones (Mediterranean climate, semi-arid climate, arid climate and extreme arid climate) defined by the Israel Meteorological Service [41] and based on the Kopen classification [42]. All these zones are characterized by long, hot, rainless summers and relatively short, cool, rainy winters. The rain period in the region takes place across the months of October–May [43]. The climate conditions within each zone of the study region vary locally by altitude, latitude, and the proximity to the Mediterranean. Summers are very humid along the Mediterranean coast but dry in the Rift Valley and the Negev Desert. The terrain also varies widely from the deepest point in the Jordan Rift Valley (− 430 m below sea level).

The study population included all singleton term births in Soroka University Medical Center (SUMC) during October 2004 – December 2013, residing in the southern district of Israel and insured by Clalit Health Services, the largest health care provider in Israel, covering approximately 70% of a population of 730,000 residents in the area [44, 45]. The rest of the population in the area is covered similarly by one of other three healthcare organizations acting under the national health act, and each resident is free to choose his/her provider among these four providers and move from one to another, with very few limitations. SUMC is a tertiary hospital in southern Israel, which receives most birth referrals in its area (Fig. 1).

Maternal and birth data were routinely collected in the Admission-Transfer-Discharge SUMC computerized database. The study population contained 56,184 singleton term births, from which we excluded subjects with missing Ta (*N* = 23) or birth weight (*N* = 9) and subjects with very extreme Ta (> 3 standard deviations, *N* = 11), leaving 56,141 subjects for analysis. tLBW was defined as birth weight < 2500 g, and SGA was defined as birth weight < 10th percentile (in Israel), according to newborn sex and gestational age at delivery [46].

### Exposure data

Air temperature: Predicted 24 h mean Air temperature (Ta) exposure data were generated by a novel hybrid spatio-temporally resolved prediction model [40]. Briefly, in these prediction model we used mixed effects models to first calibrate earth observation satellite surface temperature (Ts) data to daily mean Ta measurements from ground monitors, regressing Ta measurements against day-specific random intercepts, fixed and random Ts slopes and several spatial and temporal predictors (normalized difference vegetation index, percent urban and elevation). Then to capture the ability of neighboring cells to fill in the cells with missing Ts values, we regressed the Ta predicted from the first stage model against the mean of the Ta measurements on that day from interpolated inverse distance weighted models, discretely for each grid cell. Our model performance was excellent for both days with available Ts and days without Ts observations (cross-validated R^2^ results: 0.966–0.986). To estimate 24 h mean Ta exposure at each mother’s residence during pregnancy, we spatially joined each mother’s residence to the corresponding Ta grid cell centroid (1 km*1 km resolution) into which it fell (Fig. 1). Ta Exposure history was calculated by averaging mean daily Ta over the entire pregnancy (calculated by exact birth date and gestational age at birth for each pregnancy) and for each trimester. In general, studies in environmental epidemiology looking at acute events (such as heat waves) and the associations with health outcomes focus on acute exposure windows. This is partly due to the difficulty of detangling these acute effects from the long-term effect. In line with such previous studies [32], we decided to focus on the chronic/long term exposure. It should be noted that acute heat wave events are still included in chronic exposure moving averages and thus accounted for in the model.

Air pollution: Daily average particulate matter < 2.5 μm (PM_2.5_) concentrations were assessed using a hybrid satellite based model incorporating daily satellite remote sensing data at 1 × 1 km spatial resolution [47]. Briefly, we used an algorithm developed by NASA Multi-Angle Implementation to Atmospheric Correction, which provides aerosol optical depth (AOD) data in a high resolution. Using mixed models, we regressed daily PM_2.5_ mass concentration from the Ministry of Environmental Protection monitoring sites against AOD, traditional land use regression temporal and spatial predictors to generate daily high resolution PM_2.5_ estimations. More in depth details can be found in Kloog 2015 [47].

#### Statistical methods

For initial analyses, we used logistic regression generalized additive models with either tLBW or SGA as the dependent variable, modeling entire pregnancy Ta using a thin plate regression spline (allowing for non-linear associations). The analysis was adjusted for seasonality and time trend using categorical variables for calendar month and year, PM_2.5_, maternal age at birth (continuous, squared), gravidity, parity, ethnicity (Jewish / non-Jewish), newborn sex, census-level poverty index (a categorical variable with 20 levels, representing socio-economic level) and population density (based on the population residing in the same small-statistical-area). The two latter variables were defined and calculated by the Israel Central Bureau of Statistics [48].

Further analyses used logistic regression generalized linear models with either tLBW or SGA as the dependent variable, modeling entire pregnancy Ta using a categorical variable and each of the two extreme Ta quartiles with the two middle quartiles, adjusting for the same covariates as described above. Possible effect modification by ethnicity or urbanity were tested by adding interaction terms to these models. Trimester-specific Ta exposures were modeled similarly, in a mutually adjusted model that included the exposure in each trimester as a separate variable, in order to avoid bias from auto-correlation among Ta trimester values [49].

In a sensitivity analysis, we fit a model that included only those newborns for whom addresses were geocoded at the house or street level (*N* = 18,412). Statistical analyses were performed in R statistical software version 3.3.2 (R Foundation for Statistical Computing, Vienna, Austria) with general additive models using package mgcv [50]. A priory level of significance was 0.05 (two-sided).

## Results

The study population is described in Table 1. Of the 56,141 singleton term births included in our final dataset for analysis, 51% were males and 40% were Jewish. The mean birth weight was 3272 g (SD: 430 g), with 1716 (3.1%) cases of tLBW and 8634 (15.4%) cases of SGA. The average and the median daily Ta across the entire pregnancy were 19.9 (SD: 1.77, range: 14.6–24.9) degrees centigrade. Spearman correlation among trimester-specific Ta were: *r* = 0.048, *r* = − 0.9 and *r* = − 0.013 for pairs of trimesters 1–2, 2–3 and 1–3 respectively.

**Table 1:** Characteristics of the Study Population (*N* = 56,141), Southern Israel, 2004–2013

Characteristic	% (Number) / Mean (SD)
Ethnicity	
Jewish	40% (22,419)
Non-Jewish	60% (33,722)
Female gender	49% (27,594)
SGA	15.4% (8634)
tLBW	3.1% (1716)
Maternal age (years)	
≤ 20	4.7% (2611)
20–29	50.0% (27,929)
30–34	26.3% (14,711)
35–39	14.7% (8206)
> 39	4.5% (2520)
Urban	62.4% (35,24)
PM_2.5_ (microgram/m^3^)	20.9 (1.6)
Poverty index (1–20)	5.6 (4.4)
Birth weight (g)	3272 (430)
Gravidity	3.9 (2.8)
Parity	3.4 (2.4)

**Fig. 2:**
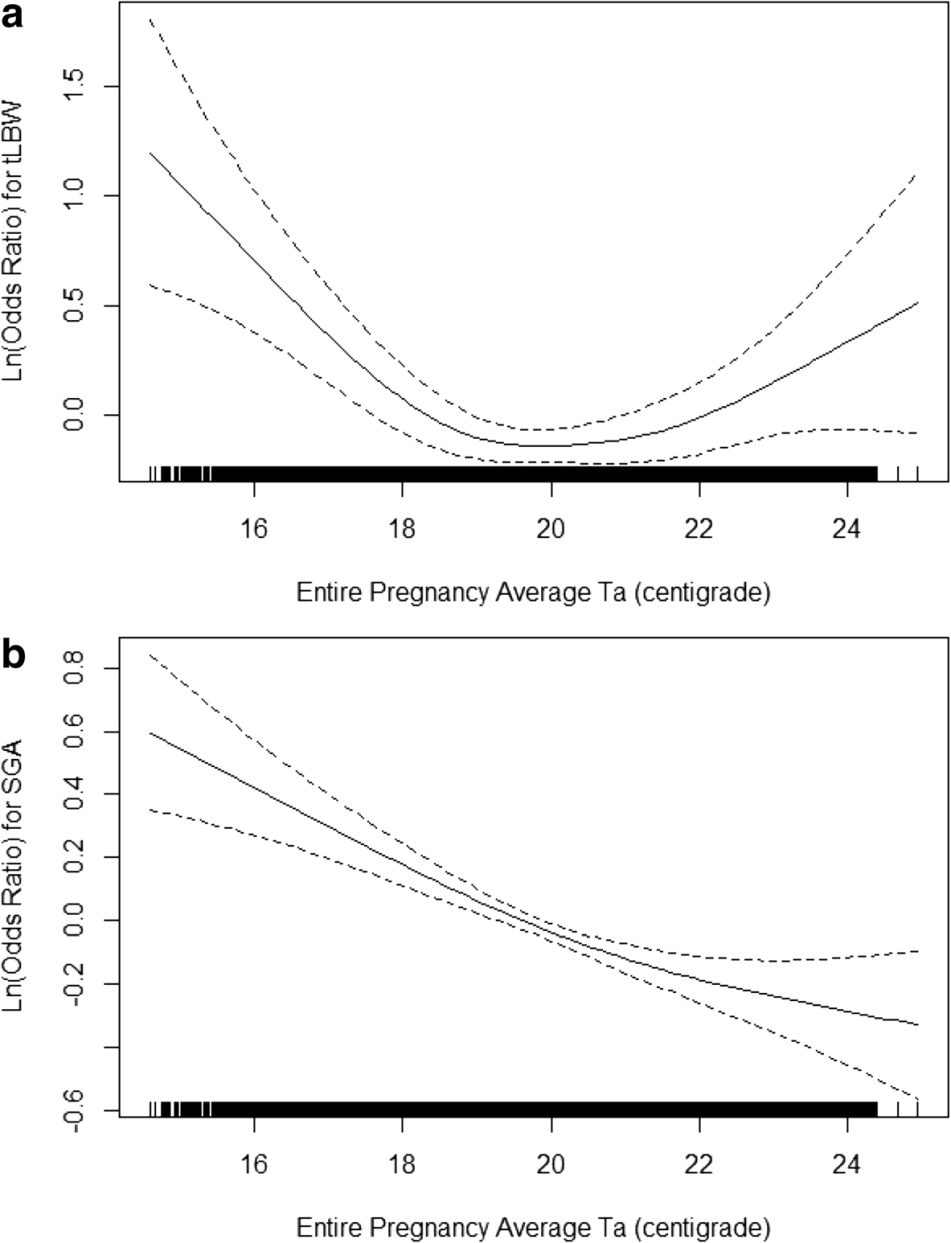
Association between Ambient Air Temperature, tLBW (panel **a**) and SGA (panel **b**) in Term Singleton Infants in Southern Israel, 2004–2013. Ta = Entire pregnancy average ambient air temperature; tLBW = term low birth weight; SGA = small for gestational age. Dashed lines around the solid line represents 95% confidence intervals. Black vertical lines along the x-axis represent a “rug” plot showing all data points. Associations were modeled using generalized additive models, adjusted for calendar month, particulate matter < 2.5 μm (PM_2.5_), year of birth, maternal age at birth, gravidity, parity, ethnicity, newborn sex, census-level poverty index and population density

Generalized additive logistic regression model revealed a significant (*p* < 0.001), nonlinear (estimated degrees of freedom = 3.2), non-monotonic association between entire pregnancy average Ta and tLBW, with both low and high temperatures associated with higher risk in comparison to intermediate temperatures, although only on low Ta was the association significant (Fig. 2a). A similar model for SGA as the dependent variable resulted in a decreasing, almost linear (estimated degrees of freedom = 2.2) curve between Ta and SGA (p < 0.001) (Fig. 2b).

We further categorized the entire pregnancy average Ta into quartiles and compared the lowest Ta quartile (Ta = < 18.5) and the highest Ta quartile (Ta > =21.3) to the two intermediate quartiles (18.5 < Ta < 21.3) using logistic regression models adjusted for the same covariates as above. The lowest Ta quartile was associated with higher risk of tLBW (odds ratio [OR] = 1.33, 95%CI 1.11–1.58) while the highest Ta quartile was not significantly associated with tLBW (OR = 1.17, 95%CI 0.99–1.38), in comparison to the two intermediate quartiles. When analyzing SGA as the dependent variable, the lowest Ta quartile was associated with significantly higher risk of SGA (OR = 1.18, 95%CI 1.09–1.29) while the highest quartile was associated with significantly lower risk of SGA (OR = 0.91, 95%CI 0.84–0.99) in comparison to the two intermediate quartiles (Table 2).

**Table 2:** Associations between Entire Pregnancy Average Ta, tLBW and SGA in Singleton Term Infants, Southern Israel, 2004–2013

Outcome	Ta Exposure Category	Odds Ratio (95% CI)
tLBW	Lowest quartile (Ta = < 18.5)	1.33 (1.11–1.58)
	Intermediate quartiles	Reference
	Highest quartile (Ta > =21.3)	1.17 (0.99–1.38)
SGA	Lowest quartile (Ta = < 18.5)	1.18 (1.09–1.29)
	Intermediate quartiles	Reference
	Highest quartile (Ta > =21.3)	0.91 (0.84–0.99)

*Ta* Average ambient air temperature, *tLBW* term low birth weight, *SGA* small for gestational age. Odds ratios were adjusted for calendar month, year of birth, particulate matter < 2.5 μm (PM_2.5_), maternal age at birth, gravidity, parity, ethnicity, newborn sex, census-level poverty index and population density

When examining associations among tLBW, SGA and trimester-specific Ta exposures, we found decreased risk of SGA with high Ta quartile during the 1st trimester (OR = 0.82, 95%CI 0.73–0.92), and increased risk for SGA with low Ta quartile during the 3rd trimester (OR = 1.17, 95%CI 1.05–1.31), while all other associations were not statistically significant (Table 3).

**Table 3:** Associations between Mutually-Adjusted Trimester-specific Ta, tLBW and SGA in Singleton Term Infants, Southern Israel, 2004–2013

Outcome	Ta Exposure Category	1st trimester	2nd trimester	3rd trimester
tLBW	Lowest quartile^a^	0.92 (0.75–1.13)	0.94 (0.74–1.18)	1.17 (0.92–1.49)
	Intermediate quartiles	Reference	Reference	Reference
	Highest quartile^a^	1.03 (0.82–1.31)	1.14 (0.92–1.41)	1.02 (0.92–1.12)
SGA	Lowest quartile^a^	1.02 (0.92–1.12)	0.95 (0.85–1.06)	1.17 (1.05–1.31)
	Intermediate quartiles	Reference	Reference	Reference
	Highest quartile^a^	0.82 (0.73–0.92)	0.94 (0.85–1.04)	0.95 (0.85–1.05)

^a^Exposure quartiles were calculated for each trimester separately. 1st trimester: lowest: < 14.9, Highest: > 24.1. 2nd and 3rd trimesters: lowest: < 15.6, Highest: > 24.7. (All values are in centigrade)

*Ta* Average ambient air temperature, *tLBW* term low birth weight, *SGA* small for gestational age. Trimester-specific exposures were adjusted for calendar month, year of birth, particulate matter < 2.5 μm (PM_2.5_), maternal age at birth, gravidity, parity, ethnicity, newborn sex, census-level poverty index and population density, and mutually adjusted for each other

In a sensitivity analysis for the entire pregnancy average Ta, including only those newborns whose addresses were geocoded at the building or the street level (*N* = 18,412), the lowest Ta quartile showed a stronger association (OR = 1.48, 95%CI 1.06–2.06), and the highest Ta quartile was still not associated (OR = 1.01, 95%CI 0.74–1.39) with tLBW, in comparison with the two intermediate quartiles. When examining Ta quartiles association with SGA in this sub-population, the associations were similar to the primary analyses but not statistically significant (OR = 1.09, 95%CI 0.94–1.28 and OR = 0.93, 95%CI 0.80–1.08 for the lowest and the highest Ta quartiles, respectively). When adding an interaction term to the main models to test for possible effect modification by ethnicity, we did not find any significant interaction between Ta and ethnicity (*p* > 0.45 and *p* > 0.2 for LBW and SGA, respectively). Similarly, no significant interaction with urbanity was found (p > 0.2 for all models).

## Discussion

In the presented study, we examined the association of Ta with tLBW and SGA in a study of term singleton births in Southern Israel between 2004 and 2013. Using our novel exposure model, we were able to assign exposure to almost all subjects with substantially reduced exposure misclassification compared to using monitored data. We found that lower Ta was associated with increased risk of tLBW and SGA, and higher Ta was associated with decreased risk of SGA in this population. When examining trimester-specific Ta, the association with higher Ta was restricted to the 1st trimester, while the association with lower Ta was restricted to the 3rd trimester. In other words, higher than usual Ta in early phases of the pregnancy seems to be protective against SGA in term infants, and lower than usual Ta in the late phases of pregnancy may be a risk factor of SGA in our study population.

Biological mechanisms that could explain a possible effect of temperature on tLBW and SGA have been rarely investigated, but several potential hypotheses exist. Some studies suggest that variation in temperature and humidity, especially at high temperature and humidity levels, can increase stress on cardiovascular function [51], which, in pregnancy, is already highly solicited. On the other hand, our results suggest higher risk of SGA in term infants with lower than average Ta, but not with higher Ta. The second and third trimesters are important times for fetal growth [52]. As extreme temperature may affect uterine blood flow and placental exchange necessary for fetal growth [53], disruption to the mechanism needed for proper growth during these time windows would have the greatest impact.

The results of our study regarding tLBW are quite consistent with those of a recent study from California, with very similar U-shape curves describing associations between apparent temperature and risk of tLBW [19]. Another recent large American study of participants from the consortium on safe labor, with almost 200,000 term infants, found both extreme high and extreme low Ta to be associated with risk of tLBW [31]. This study, however, used more extreme Ta cutoffs (<5th and > 95th percentiles) than we did. Entire pregnancy Ta was not significantly associated with SGA (without limiting to term infants) in that study, and colder Ta during the 3rd trimester were associated with lower risk of SGA, in contrast to our findings. These inconsistencies among the studies may be a result of: 1) including preterm infants when analyzing SGA; 2) differences in the populations studied; and 3) the different range of Ta in Southern Israel in comparison the colder environments inspected in other studies.

Another factor that may explain differences among study results is differences in exposure misclassification, introduced by different Ta exposure assessment methods. Stronger non-differential misclassification, as expected when using lower spatial resolution in exposure assessment, is expected to bias the estimate towards the null. Since temperature can vary greatly both spatially and temporally, the use of simple models or even temperature from sparse monitor networks and relatively large hospital capture areas can introduce considerable measurement error. Another point to note is that this lack of robust spatially resolved daily Ta exposures restricts these studies to populations surrounding monitoring stations, which may not be representative of the population as a whole. On the other hand, urbanity did not modify our effect estimates significantly, suggesting that excluding rural populations may limit sample size, but not necessarily bias effect estimates.

Our study also detected decreased risk of SGA in term infants exposed to higher Ta levels during the 1st trimester. This finding is novel and requires inspection and reproduction in other cohorts as well. We are currently not aware of biological mechanisms that may explain this finding, but it may be related to other exposures, which are a result of pregnant women changing their behavior in response to high ambient temperatures, such as differences in physical activity or time spent indoors. Previous studies that examined 1st trimester exposures did not result in a similar finding, but their published results were also not adjusted for exposures during the other trimesters [19, 31]. This adjustment is important in order to avoid bias resulting from correlation of the exposure among various exposure windows, as demonstrated using simulations in the case of air pollution [49].

Comparison with the results of other studies of Ta and birth weight is more complicated: Most studies took a different analytical approach, examining the associations with birth weight as a continuous variable, unlike the approach we took, concentrating on clinical categories (tLBW and SGA) which are known predictors of health and disease. From the studies that did examine LBW we found 3 ecological studies [54–56] and one retrospective cohort [32] . From these, only one of the ecological studies was restricted to term births like the current study, and found no significant associations of LBW with or cold waves [56]. However, the differences in study design (ecological vs cohort) and the limited sample size in that study (approximately 1500 births) can account for the difference in the results.

Another thing to note is that there are clear and significant changes in average Ta in Israel in recent decades. Since the 1970s, average annual temperature increased by close to 1 °C [57]. These trends, if persist, have serious environmental and health implications in the future. However, we believe that they did not impact our study results, which are based on births during 2004–2013 and compare stronger Ta exposure contrasts (e.g. 14.6–18.5 and 18.5–21.3 for the two lowest comparison groups).

There are several limitations in the present study. First, the spatial resolution of the exposure was 1 × 1 km and exposures were assigned based on best-available residential address geolocation. We are planning to improve our hybrid models using new available satellites and machine learning methodologies to generate higher spatial resolution data reaching 100 m, which will further reduce exposure error. Other limitations with the SUMC database include the lack of some health and personal level data such as maternal weight and height, chronic hypertension, preeclampsia or gestational hypertension, maternal smoking and physical activity, which were not available. On the other hand, we do not have a reason to think that these characteristics are a common cause for both ambient Ta and tLBW or SGA, and therefore they should not be a source of concerns of residual confounding.

## Conclusion

Our findings suggest that lower pregnancy Ta may increase the risk of tLBW and SGA, and higher pregnancy Ta may decrease the risk of SGA in singleton term infants in southern Israel. Future studies may examine associations of Ta with birth weight in preterm infants and examine possible mechanisms of effects of Ta on fetal growth.

## Abbreviations

AOD: Aerosol optical depth
HR: Hazard ratio
LBW: Low birth weight
PM_2.5_: Particulate matter < 2.5 μm
SGA: Small for gestational age
SUMC: Soroka University Medical Center
Ta: Ambient air temperature
tLBW: Term low birth weight
Ts: Surface temperature
